# Efficacy and Safety of Trans-nasal Sphenoid Ganglion Block in Obstetric Patients With Post-dural Puncture Headache: A Randomized Study

**DOI:** 10.7759/cureus.20387

**Published:** 2021-12-13

**Authors:** Nazia Nazir, Anupriya Saxena, Unnati Asthana

**Affiliations:** 1 Anesthesiology and Critical Care, Government Institute of Medical Sciences, Greater Noida, IND; 2 Anesthesiology and Critical Care, Employees' State Insurance (ESI) Dental College and Hospital, New Delhi, IND

**Keywords:** visual analog scale, headache, epidural blood patch, post-dural puncture headache, sphenopalatine ganglion block

## Abstract

Introduction

This study evaluated the efficacy and safety of two methods to achieve a trans-nasal sphenoid ganglion (SPG) block in obstetric patients for treating a post-dural puncture headache was evaluated.

Methods

In this prospective single-blinded randomized study, 20 enrolled patients were divided into two groups: group 1 (n=10) received SPG block via the applicator method and group 2 (n=10) by the nasal spray technique. The reduction in the pain score, number of patients requiring rescue analgesia with time to first analgesic request, repeat procedure required, and any adverse event were recorded.

Results

Patients in both groups were comparable with respect to the baseline characteristics. After the SPG block, the patients in group 1 had a significant reduction in the visual analog score (VAS) as compared to group 2 in the first 24 hours (P<0.001). Thereafter, the pain scores were comparable between the groups till discharge. Only one patient in group 1 required rescue analgesia as against six in group 2 (P= 0.02, OR= 13.5). The procedure was repeated in 10% of patients in group 1 and 30% of patients in group 2 (P= 0.26, OR= 3.85). On intragroup comparison, both groups revealed a significant reduction in pain from the baseline after the block (P<0.001).

Conclusion

The trans-nasal SPG block is a minimally invasive treatment option for post-dural puncture headache (PDPH) and avoids the need for more invasive treatment techniques. Among the two approaches of a trans-nasal SPG block, the applicator technique results in better pain relief.

## Introduction

Post-dural puncture headache (PDPH), an undesirable complication of neuraxial anesthesia, has been a challenge for anesthesiologists. International Classification of Headache Disorders (ICHD-3) has defined PDPH as a headache attributed to low cerebrospinal fluid (CSF) pressure (intracranial hypotension) resulting from loss of CSF that exceeds spinal fluid production [1]. Risk factors for PDPH include female gender, pregnancy, younger age, and the use of a cutting or larger bore spinal needle [2]. Patients' suffering is exacerbated by PDPH, as is the duration of stay and overall expense of care in the hospital.

Management of PDPH is challenging; several treatment options are available, ranging from conservative (supine position, hydration, an abdominal binder, analgesics, caffeine, sumatriptan, and laxatives) to more invasive approaches for persistent headaches (autologous epidural blood patch) [3].

An autologous epidural blood patch is considered the gold standard in the treatment of persistent PDPH with an efficacy of 75% [3]. It is an invasive technique associated with complications like meningitis, arachnoiditis, seizures, loss of hearing or vision, radicular pain, subdural hematoma, and neural deficits [4].

Recently, some researchers have reported the effectiveness of a sphenopalatine ganglion block (SPG) in the treatment of PDPH [5-6]. There is a trans-nasal, trans-oral, sub-zygomatic, and lateral infratemporal approach for this block [3]. The trans-nasal approach to SPG block is minimally invasive, office-based, and safe [3].

This study was planned to study the efficacy and safety of two trans-nasal approaches to achieve sphenopalatine ganglion block for the treatment of PDPH in obstetric patients following neuraxial anesthesia. We hypothesized that a simple diffusion of the drug by an easier method like nasal spray results in as effective an SPG block as by the applicator method.

## Materials and methods

Study design

The present study was a single-blind prospective study, conducted over a period of four years from July 2016 to December 2020 in a tertiary care hospital. The study was approved by the institutional ethics committee (IEC/2016/76-A/03), and the participants provided written informed consent before inclusion.

Sample size

We classified the patients with PDPH to have an average initial visual analog score (VAS) of eight in a sitting position. With a standard deviation of two, we treated a reduction in VAS by three to be clinically relevant. With a 95% confidence interval and 80% power, the minimum sample size required to obtain statistically significant results with a P-value of 0.05, was seven for each intervention group with 1:1 enrolment. Considering dropout or refusal to repeat the procedure, we took a sample of 10 participants in each group.

Study population

Eligible patients were 20 obstetric patients, of the American Society of Anesthesiologists I & II (aged ≥ 18 yrs), presenting with PDPH within five days of neuraxial anesthesia. The headache should have persisted for at least two days after dural puncture and must have been intractable to conservative treatment with fluids, caffeine, paracetamol, and other nonopioid analgesics. Patients were excluded if they refused to give consent, had known coagulopathy/ nasal septal deviation/nasal bleeding, had a history of allergy to lidocaine, or had received opioids less than 12 hours before study inclusion.

Treatment allocation 

**Figure 1:**
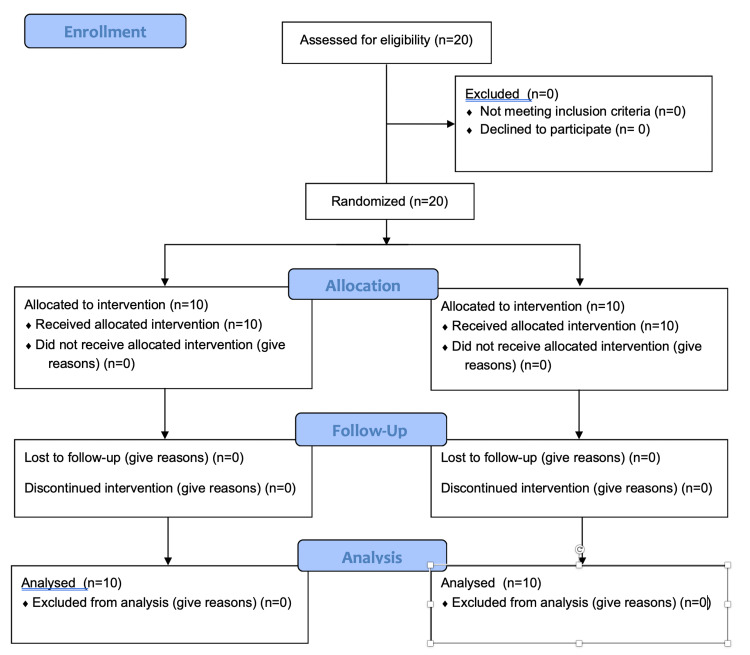
CONSORT Flow Diagram

Randomization was based on a computer-generated randomization list. Patients were randomized to receive an SPG block by either of the two methods (applicator method and nasal spray method) (Figure 1).

In both groups, the procedure was carried out in the operation theater to maintain strict asepsis by an experienced anesthetist (with more than eight years of experience). Baseline heart rate, blood pressure, and pain score by VAS were measured. No sedative was given prior to the procedure. The patients were asked to blow out each nostril and then lay supine with a small shoulder roll so that their neck is extended and their nostrils pointed in an upward direction. Intranasal decongestant was sprayed once into each nostril preemptively to minimize bleeding.

**Figure 2:**
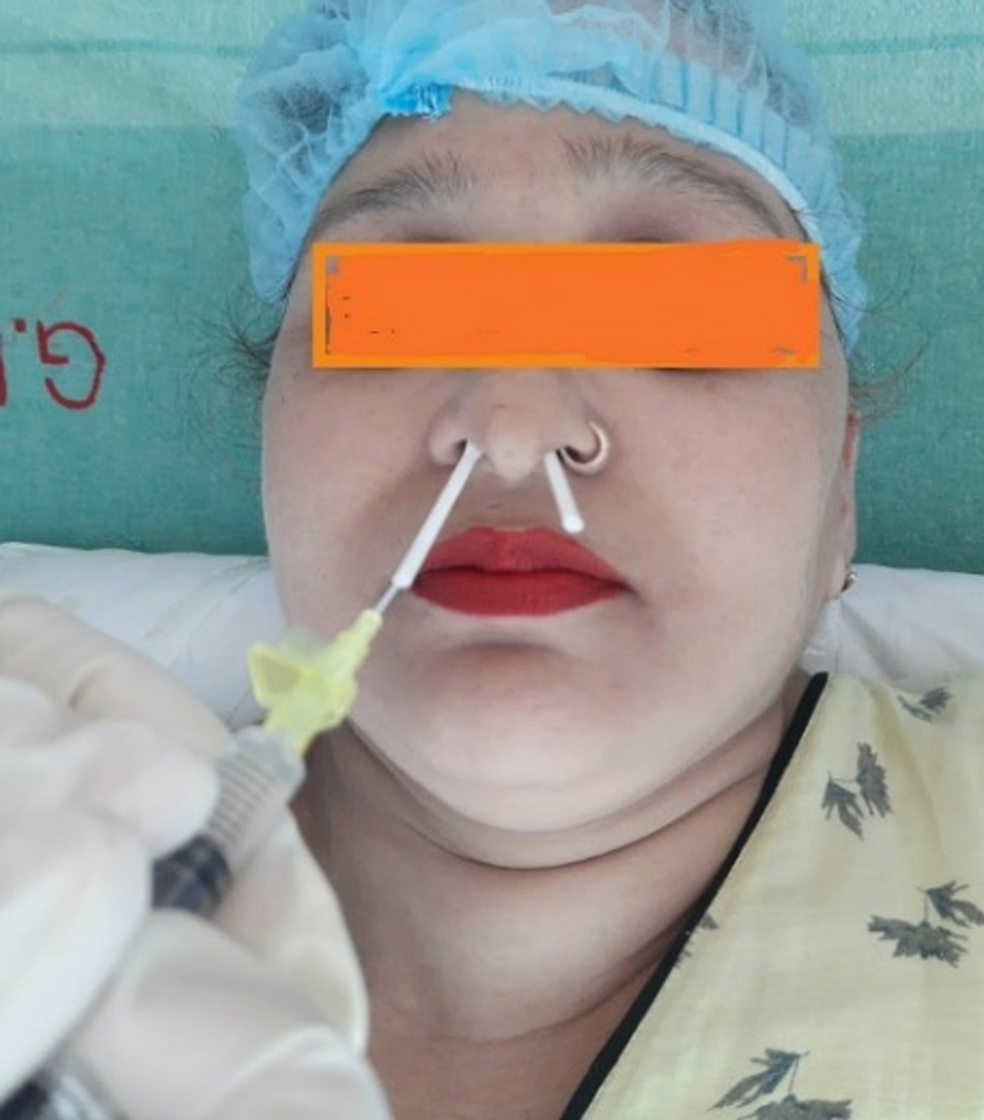
Trans-Nasal Sphenopalatine Ganglion Block by Applicator Method

In Group 1, the sphenopalatine ganglion block was performed by inserting long cotton-tipped applicators saturated in 2% viscous lidocaine (Xylocaine® Viscous; Zydus Healthcare, Ahmedabad, India) into both the nostrils until properly seated in the posterior nasopharynx (Figure 2).

The applicator was left in place for five minutes and then 1 ml of 2% lidocaine (40 mg) (LOX* 2%) (safe dose of lidocaine 4.5 mg/kg) was administered down the plastic hollow shaft of each applicator via an angiocatheter (until the patients felt the solution in the back of their throats). The applicators were kept in place for 10 more minutes and then removed.

In Group 2, the patients were asked to take a deep breath, hold it, and exhale when signaled to do so. At the height of inspiration, two puffs of lidocaine 10% (40 mg) (LOX* 10% spray) were administered into one nostril aimed slightly medially and inferiorly. They were advised to keep their eyes closed during spray administration. The patients were signaled to exhale after a few seconds. After the patient’s condition settled down (stable vital parameters), the same process was repeated for the other nostril.

In both the groups after 10 minutes of procedure, the patients were asked to sit up and the pain was assessed using the visual analog score (VAS), (0 - no pain to 10 - worst pain imaginable) [7]. The VAS was recorded before the procedure and then at 30 min, 1, 6, 12, and 24 h on the day of SPG block and then once daily till the day of discharge. The patients who failed to achieve adequate pain relief with VAS ≥8 were offered rescue SPG block by the same anesthesiologist who performed the first procedure to avoid any technical bias.

Patients having VAS≥5 were administered rescue analgesia with injection diclofenac 75 mg intravenously. Heart rate, mean arterial pressure, and post-treatment complications, such as throat numbness and nasal bleeding, were also recorded along with VAS scores. Patients who developed any throat numbness were advised not to eat or drink anything for 12 h to avoid the risk of choking.

Outcome

The primary outcome of this study was to compare the efficacy of a trans-nasal SPG block for the treatment of PDPH by the two methods as assessed by reduction in the pain level with VAS in the upright position. 

Secondary outcome measures were the number of patients requiring rescue analgesia with time to the first analgesic request, repeat procedure required with the timing of repeat procedure, development of an adverse event related to drug or procedure, i.e., changes in blood pressure and heart rate, numbness in the throat or nasal bleeding.

Statistical analysis

The data collected from 20 study participants were entered in Microsoft Excel version 2016 (Microsoft Corporation, Redmond, WA). Quantitative data were expressed in mean and standard deviation. Qualitative data were expressed in proportion and percentages. Independent t-tests were applied to compare the means of quantitative data and the chi-square test was used to compare categorical data. P-value < 0.05 was considered significant. Graphs were formed using Excel software.

## Results

A total of 20 patients were enrolled and all patients completed the study. The two study groups were comparable with respect to the baseline characteristics (Table 1).

**Table 1:** Baseline parameters of patients given a sphenopalatine ganglion block Data are reported as number (%) as appropriate.

Parameter	Group 1 (n=10)	Group 2 (n=10)
Mean age (yr)	28	27.5
Previous history of post-dural puncture headache	0	0
History of frequent headaches	1 (10)	0
Anxiety	2 (20)	1 (10)
Comorbidity (Hypertension)	0	1 (10)

Measurement of pain

The baseline VAS scores were comparable between the two groups with a P-value of 0.407 (Table 2).

**Table 2:** Comparison of pain score, time of the analgesic request, and time of the repeat procedure between the groups Data are reported as mean and standard deviation and number (%) as appropriate. *VAS: Visual Analog Scale; **DOD: Day of Discharge

Parameter	Group 1 (n=10) Mean±S.D	Group 2 (n=10) Mean±S.D	Sig. (2-tailed)	95% Confidence Interval of the Difference	
				Lower	Upper
VAS* Baseline	8.70 ± 0.483	8.90 ± 0.568	0.407	-.695	.295
VAS 30min	3.80 ± 0.919	5.20 ± 0.632	0.001	-2.14	-.659
VAS 1hr	3.50 ± 0.527	4.80 ± 1.033	0.002	-2.07	-.530
VAS 6hr	3.70 ± 1.567	6.20 ± 1.476	0.002	-3.93	-1.070
VAS 12hr	3.81 ± 0.483	4.90 ± 1.287	0.013	-2.11	-.287
VAS 24hr	3.75 ± 0.483	4.80 ± 0.632	0.000	-1.62	-.571
VAS DOD**	2.40 ± 1.40	3.20 ± 0.60	0.229	-0.41	1.611
Time to first rescue analgesia	210 ± 0.0	145.000 ± 166.94	0.552	-578.52	348.52
Time to repeat the procedure	440	480 ± 207.84	0.667	-1152.63	912.63

**Figure 3:**
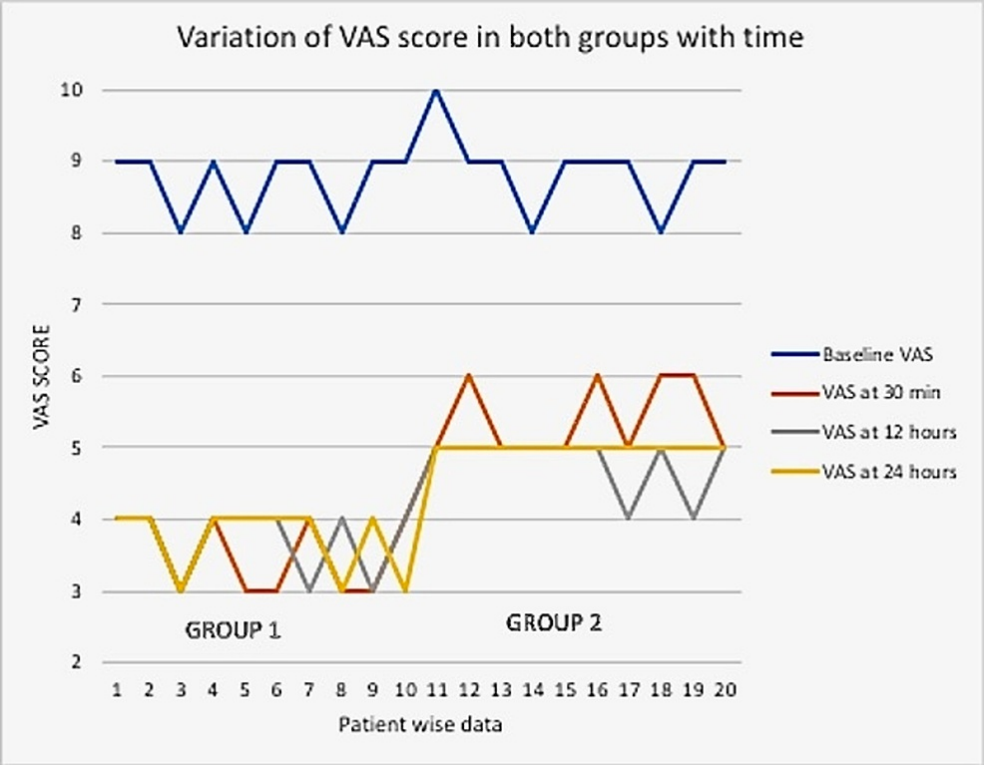
Line diagram showing variation in VAS score in both groups with time VAS: Visual Analog Scale

The patients in group 1 had a significant reduction in VAS as compared to group 2 at all time points of measurement during the first 24 hours. Thereafter, the pain scores were comparable between the groups till the day of discharge (Table 2, Figure 3).

In both groups, the VAS at 24 h was significantly reduced when compared to baseline VAS, reflecting a reduction in pain from the baseline after both the methods of the block (Table 3).

**Table 3:** Intragroup variation in VAS score Data are reported as mean and standard deviation. * Baseline VAS (Visual Analog Scale)

Group	BL VAS* (Mean±SD)	VAS at 24h (Mean±SD)	Sig. (2-tailed)	95% Confidence Interval of the Difference	
				Lower	Upper
1	8.70 ± 0.48	3.75 ±0.48	0.00	4.49	5.40
2	8.90± 0.56	4.80± 0.63	0.00	3.53	4.66

Rescue medication

Only one patient in group 1 required rescue analgesia as against six in group 2 (P=0.02, OR=13.5, CI 1.2-152.01). The procedure was repeated in 10% of patients in group 1 and 30% of patients in group 2 (P=0.26, OR=3.85, CI 0.32-45.57). No additional requirement of rescue analgesia or repeat procedure was required in either of the groups after the first 24 h of the procedure till the discharge of the patients (Table 4).

**Table 4:** Comparison of frequency of repeat procedure, rescue analgesia, and complications between the groups Data are reported as numbers (%). * Confidence interval

Parameter	Group 1	Group 2	P-value	Odds ratio (C.I.*)
Repeat procedure required	1(10)	3(30)	0.26	3.85 (0.32-45.57)
Rescue analgesia required	1(10)	6(60)	0.02	13.5 (1.20-152.21)
Throat numbness	3(30)	2(20)	0.60	0.58 (0.07-4.56)
Nasal bleeding	1(10)	0	0.30	0.47 (0.29-0.76)

The time of first rescue analgesia was 210 ± 0.0 min in group 1 as compared to 145± 166.94 min in group 2 (P=0.552) (Table 2).

Adverse events

**Figure 4:**
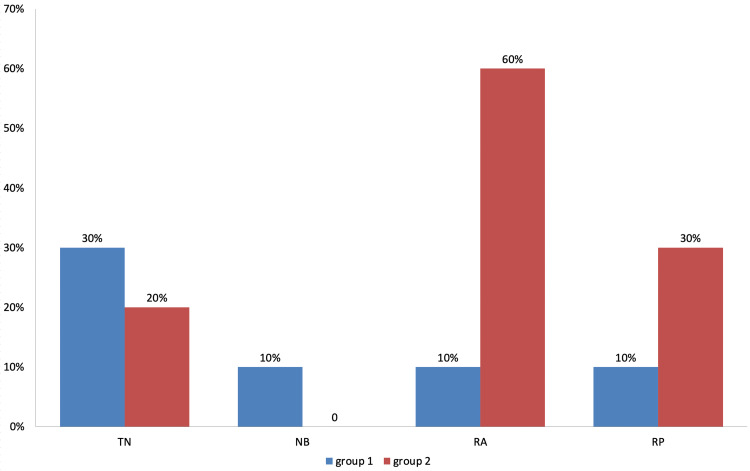
Comparison of complications in both groups TN: Throat Numbness; NB: Nasal Bleed; RA: Rescue Analgesia; RP: Repeat Procedure

Complications like throat numbness (due to swallowing a small amount of the LA) and nasal bleed were observed to be slightly higher in group 1, although the difference was statistically insignificant (Table 4, Figure 4).

The groups did not show any statistically significant difference in comparison of heart rate and mean arterial pressure in the baseline and post-procedure values at any point in time (Tables 5-6).

**Table 5:** Heart rate variation among the two groups

Time Points	Group 1	Group 2	P value
Baseline	93.00± 2.16	92.40±1.26	0.457
30 minutes	80.90±2.60	83.0± 2.16	0.065
1 hour	81.10±1.96	82.70±1.94	0.084
6 hour	80.50±2.46	81.40±1.64	0.349
24 hour	78.60±1.89	80.20±1.98	0.821
Day of discharge	75.90±1.99	78.45±2.10	0.266

**Table 6:** Mean arterial blood pressure variation among the two groups

Time Points	Group 1 (mean±SD)	Group 2 (mean±SD)	P-value
Baseline	82.30±1.49	82.50± 1.26	0.75
30 minutes	78.70±0.82	78.30± 0.94	0.32
1 hour	77.60±1.50	78.30±1.33	0.28
6 hour	77.90±0.58	78.70±1.05	0.05
24 hour	78.80±0.91	78.70±1.33	0.84
Day of discharge	80.76±2.33	82.00±1.5	0.174

## Discussion

The sphenopalatine ganglion is a parasympathetic ganglion lying posterior to the middle turbinate surrounded by mucous membrane [3]. The sphenopalatine ganglion (SPG) block was traditionally used to treat headaches and atypical facial pain [8-9]. It has shown promising results in treating post-dural puncture headache (PDPH) with minimal side effects as compared to other invasive methods used for PDPH treatment [10]. There is a trans-nasal, trans-oral, sub-zygomatic, and lateral infratemporal approach for this block [3].

The role of the SPG block in alleviating this headache is attributed to the fact that after a dural puncture, the CSF is continuously lost and the intracranial volume is restored by compensatory vasodilatation (Monro-Kellie doctrine) [11]. This vasodilatation is mediated by the parasympathetic activity of the neurons, which have synapses in the SPG, resulting in an excruciating headache after a dural puncture [12].

In this study, the VAS score was significantly lower in group 1 as compared to group 2 at all points of time during the first 24 hours. The majority of patients did not receive any rescue analgesia in group 1. In comparison, six patients in group 2 required rescue analgesia and the difference was statistically significant (P=0.02, OR=13.5, CI-1.20-152.21). This points to a better therapeutic outcome seen with an SPG block by the trans-nasal applicator approach as compared to the trans-nasal spray method. Various etiologies postulated for effective SPG block are mechanical stimulation of SPG, absorption of local anesthetic through the mucous membrane, the pressure created by the cotton applicator or blockade of parasympathetic mediated vasodilatation [13]. Whatever the mechanism, it has resulted in a comparatively better outcome and pain relief than the nasal spray method of SPG block. This can be due to the potential of the misplaced drug leading to a lesser concentration of drug reaching the effect site, resulting in decreased diffusion of drug across the mucous membrane and hence reduced/ineffective block in some patients. However, in both the groups there was a reduction in pain from the baseline after the block (Table 3). This signifies that the trans-nasal SPG block, whether achieved by the applicator method or nasal spray method, does have a role as a non-invasive or minimally invasive method for the management of PDPH [8,14-16]. Thus, it can be used as the first line of management, as the analgesia produced is rapid and effective in most cases. 

The incidence of adverse events was reported to be 40% in the applicator group and 20% in the spray group, but the difference was statistically insignificant. One patient in the applicator group complained of nasal discomfort and had a blood-tinged cotton applicator on removal. However, no obvious signs of bleeding or hemodynamic instability were present. The discomfort disappeared after some time without any intervention. 

An SPG block can be performed via the trans-nasal, transoral, sub-zygomatic, and lateral infratemporal approaches. However, the last three approaches are difficult, requiring sufficient training to administer the block [3]. In contrast, the trans-nasal SPG block is a simple approach [3]. The applicator method of the trans-nasal SPG block is contraindicated in patients with basilar skull fractures, local infection, or coagulation dysfunction [5]. Hence, a new management paradigm with the use of a nasal spray of a local anesthetic to achieve a trans-nasal SPG block was tried. The obvious advantage of the nasal spray method is the ease of administration. In our study, the patients were administered both types of blocks in the settings of an operation theater. The SPG block by nasal spray method has the potential to be done at the bedside or at home also after patient education, as being practiced by patients having chronic pain [17]. This can reduce the visits to the emergency department, improve patient satisfaction, and lower health care costs. However, the nasal spray was not as effective as the applicator method in reducing pain intensity (Table 2), but a significant reduction in pain from the baseline was achieved in all the patients in both groups (Table 3). Hence, the nasal spray approach needs to be studied in a larger number of patients to find the potential advantages and cost-benefit ratio.

There have been several case reports reporting the beneficial effect of a trans-nasal SPG block by the applicator method in relieving PDPH. A randomized controlled trial conducted by Jespersen et al. concluded that the pain intensity of PDPH was reduced with an SPG block [13]. However, a non-significant effect of local anesthetic in comparison to placebo in relieving PDPH was noted. Some case reports have suggested a modification for achieving an SPG block using an epidural catheter [14], nasal drops [15], and nasal spray [16]. However, a thorough literature search did not reveal a study that compared the two methods of achieving the trans-nasal SPG block used in our study.

The strength of our study was that first, we included a homogeneous population of only obstetric patients developing PDPH. Secondly, in the event of an inadequate block or breakthrough pain, a repeat procedure was performed using the same technique and the same anesthesiologist, preventing operator or technique bias. This also helped in further evaluation of the effectiveness of a particular technique.

Our study had some limitations as well. First, it was a single-blinded study, as only the person collecting the data was unaware of group allocation. The duration of analgesia could have been increased with the use of a long-acting local anesthetic like ropivacaine. However, due to the unavailability of ropivacaine spray, lignocaine spray was selected.

## Conclusions

In conclusion, we found that the administration of a trans-nasal SPG block is a simple and minimally invasive treatment option for PDPH. It avoids the need for more invasive treatment techniques. Among the two approaches of trans-nasal SPG block, the applicator method results in better pain relief. However, the nasal spray method also has some role in pain reduction, and more studies with larger sample sizes are required to further validate the results.
